# A Patient with Concurrent Legionella and COVID-19 Infection in a UK District General Hospital

**DOI:** 10.1155/2022/6289211

**Published:** 2022-11-24

**Authors:** Jie L. Tong, Michael A. Long, Peter Russell

**Affiliations:** The Princess Alexandra Hospital, Hamstel Road, Harlow, London CM20 1QX, UK

## Abstract

A 65 year-old gentleman had been brought to our Respiratory Emergency Department for patients with respiratory symptoms and a possible COVID-19 infection with a 3-day history of shortness of breath (SOB), fever, a productive cough of yellow sputum, and right-sided chest pain. The patient had received both vaccinations at the time and initially reported no travel history, although later it was revealed that he had recently stayed at a hotel. He tested positive for COVID-19 and had hyponatremia and a raised procalcitonin, indicating a bacterial infection as well. He had been initiated on our local treatment guidelines for COVID with antibiotics, guided by local hospital guidelines. An atypical pneumonia screen returned a positive result for Legionella urine antigen, and his antibiotic regime was changed accordingly. Our patient deteriorated significantly, and despite being escalated to our intensive care unit (ICU), he unfortunately passed away. Our case highlights the importance of early ICU involvement and escalation of antibiotics in cases of suspected concurrent Legionella and COVID-19 infections.

## 1. Introduction

On the horizon is a new variant of the severe acute respiratory syndrome coronavirus 2 (SARS-CoV-2), termed the “omicron” variant. Healthcare services have streamlined their approaches to the handling and management of SARS-CoV-2, its resulting disease, COVID-19, and possible complications, such as concurrent bacterial infections. Indeed, providing separate admission pathways to ensure reduction of spread, standardisation of treatment packages, and use of procalcitonin as a marker for bacterial infection are but a few adaptations to clinical practices this global health crisis has birthed.

Concurrent bacterial infections are a notable complication of SARS-CoV-2 infection [1], and studies are beginning to be published detailing the breakdown of likely pathogens [2, 3]. At the time of writing, Legionella has not been included in such studies, likely due to the rarity with which such cases are seen. As such, there are but a few case studies currently published detailing concurrent Legionella infection or “Legionnaire's Disease” (LD) and COVID-19, with guidance on this being anecdotal currently.

Our case demonstrates the need for and importance of early ICU involvement in cases of suspected concurrent Legionella and COVID-19 infections with a heavy emphasis on the likely need for multiorgan support. Early escalation of antibiotics to cover for atypical causes of pneumonia is another aspect of our case that may have changed the course of this case.

## 2. Case Presentation

A 65 year-old gentleman had been admitted to our emergency department in September 2021 with suspected community-acquired pneumonia (CAP) on a background of asthma and chronic obstructive pulmonary disease (COPD), as he is an ex-smoker with a 50-pack-year history. He had no limitation to his activities and had a regular budesonide/formoterol inhaler (200/6 two puff twice daily) alongside supplemental home oxygen and nebulised bronchodilators as required with no exacerbations for the past year. He had been double vaccinated with the Oxford–AstraZeneca ChAdOx1 nCoV-19 vaccine (AZV) [4] with his last vaccination being in May 2021. Initial history in the respiratory emergency department indicated increasing breathlessness over 3 days with fever and intermittent right shoulder pain, radiating to his back, associated with coughing and movement. He had been started on intravenous (IV) fluids and nebulisers for exacerbation of COPD as well as supplemental oxygen maintaining saturations above 94% on 15 litres and referred to the acute medical speciality. Further history revealed a productive cough of yellow sputum, but the patient stated that he had not recently travelled abroad or had any changes to his home environment. It had later been revealed that although (due to the COVID-19 pandemic) the patient had not travelled abroad, he had recently stayed at a hotel for a music festival.

On examination the patient looked unwell and short of breath; auscultation revealed bilateral coarse crackles, worse in the right upper zone as well as polyphonic wheeze, especially on the left side. Public Health England was informed the next morning, as suspected Legionella cases are notifiable in the UK.

### 2.1. Investigations

**Figure 1:**
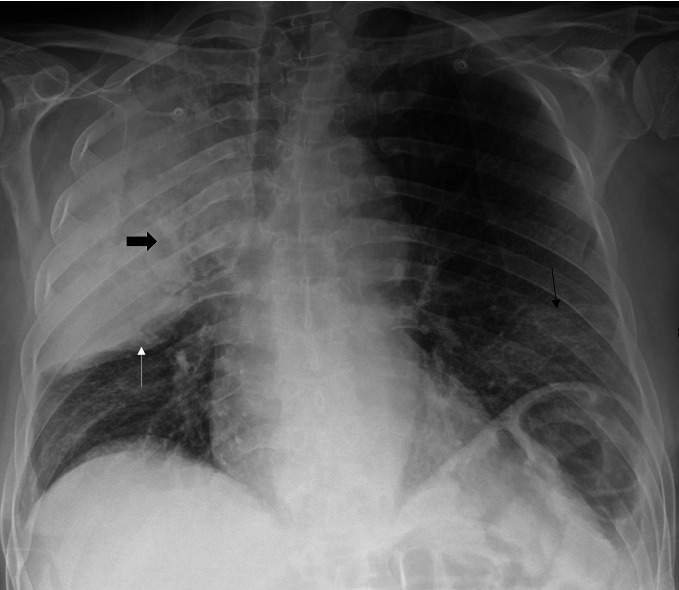
Chest X-ray. AP projection. Consolidation expanded the right upper lobe, with a mildly bulging horizontal fissure and a thin white arrow. Several small foci of cavitation exist within the consolidated RUL, one of which is indicated by the thick black arrow. Bilateral lower zone ground-glass opacities, thin black arrow on the left.

Despite being double vaccinated, our patient tested positive for SARS-CoV-2 by simple amplification-based assay (SAMBA) [5], done during triage, and a subsequent PCR test reported the following morning confirmed a positive result. A repeat was done the following day as per hospital policy when transferring from acute wards to medical wards; it remained positive. An initial arterial blood gas demonstrated Type 1 respiratory failure and hyponatremia, and an electrocardiogram showed sinus tachycardia. Initial blood results demonstrated a normal white cell count (10.0 × 10^9^/L), but with a raised neutrophil count (9.3 × 10^9^/L) and a low lymphocyte count (0.5 × 10^9^/L). There was also raised c-reactive protein (527 mg/L), ferritin (631 *μ*g/L), lactate dehydrogenase (421 IU/L), and a troponin of (9.8 ng/L). There was also a raised procalcitonin (6.8 *μ*g/L) suggesting a concurrent bacterial infection [6] and hyponatraemia (129 mmol/L). Subsequent blood results taken 4 hours later demonstrated a fall in troponin (8.1 ng/L) and a raised D-Dimer (7088 ng/ml), as shown in Table 1. His admission chest X-ray (Figure 1) demonstrates dense consolidation in the right upper lobe.

**Figure 2:**
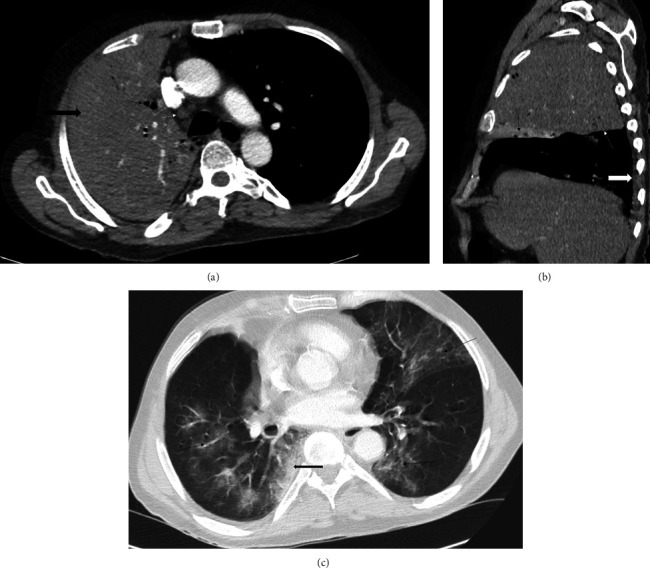
(a) Axial contrast enhanced CT image with mediastinal window settings demonstrating consolidated RUL (thick black arrow) with foci of cavitation, one of which is indicated with the thin black arrow. Mildly enlarged pretracheal-retrocaval lymph node (thin white arrow). (b) Contrast-enhanced CT reconstruction through the right hemithorax with mediastinal window settings. Expanded, consolidated RUL with depression of the oblique fissure indicated by a thin white arrow. Collapsed RML demonstrating enhancement, contrasting with the hypo-enhancing consolidated RUL (thin black arrow). Small right pleural effusion (thick white arrow). (c) Axial CT image with lung window settings demonstrating bilateral multilobar foci of ground-glass density (thin black arrows) and a focus of RLL paravertebral subpleural consolidation (thick black arrow).

Blood results taken the next day demonstrated resolution of both neutrophilia (5.6 × 10^9^/L) and hyponatremia (135 mmol/L), respectively, but ongoing lymphopenia (0.4 × 10^9^/L). An atypical pneumonia screen for Legionella and pneumococcal antigen was sent off initially sent on admission clerking by the acute medical team returned a positive Legionella urine antigen result the next morning. Blood cultures taken at time of admission and subsequently did not yield any positive results. He had a CURB65 score = 4 for severe community-acquired pneumonia. The initial sputum culture taken on admission demonstrated scant *Candida albicans* growth. A computed tomography pulmonary angiogram (CTPA) was taken to rule out pulmonary embolus (PE), the image quality was heavily affected by the other ongoing pathologies but did not demonstrate any embolus (Figure 2), instead confirming the X-ray findings, although notably now bilateral ground-glass opacities and consolidation and a small amount of right-sided pleural effusion. A subsequent culture taken on our COVID-19 positive ward was sent to our reference laboratory for specialised culture and after 11 days was reported confirming a diagnosis of Legionella pneumophila serogroup 1.

### 2.2. Treatment

Initial management centred around a combination of intravenous fluids, antibiotics (Ceftriaxone, 2 grams once daily intravenously), corticosteroids alongside supplemental oxygen, and nebulised Salbutamol and Ipratropium as per our hospital guidelines. Guided by the raised D-Dimer, he had been initiated on treatment dose enoxaparin whilst awaiting a CTPA. Despite this treatment, the patient's clinical condition deteriorated and the addition of a macrolide (Clarithromycin 500 milligrams twice daily intravenously) was decided during the following morning's consultant ward round. Following this, the ward was contacted with a presumptive positive Legionella urine antigen result, and we decided on the addition of a quinolone (Levofloxacin 500 milligrams twice daily intravenously, respectively), following guidance for treatment of Legionnaire's disease from our microbiologist and Public Health England.

Initially, the patient had been discussed with intensive care specialists and he had not been keen for escalation of noninvasive ventilation as he had not been tolerating high flow oxygen at the time.

### 2.3. Outcome

Throughout the morning, the patient's breathing became more laboured, and following his CTPA, he agreed to be escalated to the ICU for intubation and multiorgan support. In total, this patients' admission lasted 48 hours. Unfortunately, the patient went into cardiac arrest during transfer to ICU and despite cardiopulmonary resuscitation, did not survive this admission. Because of the nature of this case, a postmortem was carried out, and with the reference lab results confirming the presence of Legionella pneumophila in both urine and sputum, Legionnaire's disease was determined as the primary cause of death with COVID-19 being a secondary cause. In total, the patient was in our care for only 2 days.

## 3. Discussion

The causes of CAP are wide and varied, but in the era of COVID-19, healthcare professionals are hard pressed not to consider SARS-CoV-2 infection as the primary cause of acute presentations with cough, fever, and SOB. Nevertheless, it is of paramount importance that now more than ever we keep in mind differentials including both typical and atypical causes of CAP. Bacterial coinfections are a well-known complication of COVID-19 with protocols now in place to differentiate them from purely COVID-19. It is also worth bearing in mind other viral causes of pneumonia as we approach a post-COVID era. PE is another well-recognised complication of SARS-CoV-2 infection that we excluded [7] and, without prompt treatment, can cause sudden death in any patient.

Our patients' age and gender are identified as risk factors in COVID-19 severity [8]. His history of asthma has not yet been confirmed to be directly implicated in the outcome from COVID-19 infection [9], but it is recognised as a contributing factor to the incidence of bacterial pneumonia and by extension at increased risk of Legionnaire's disease caused by L. pneumophila.

Legionella and its associated diseases are a public health concern due to their relation to colonisation of key infrastructure (i.e., water distribution systems) and prompt thorough investigations from the relevant safety bodies. Standardised definitions of disease, surveillance, and “water safety plans” in Europe were establish in the 1980s and have thus far had a significant effect on reducing cases [10]. Disease caused by Legionella were firstly a case of Pontiac Fever in 1968 and more commonly known an outbreak of Legionnaire's Disease in Philadelphia [11]. It was later identified that Legionella caused both.

Legionella are a group of Gram-negative bacilli and a thankfully rare cause of pneumonia in the United Kingdom (UK), with around 500 cases identified last in 2019 [10]. It is most commonly contracted after exposure to the airborne bacterium from infected water distribution systems. The serogroup identified in our case was L. pneumophila serogroup 1 (sgp1) sequence type 48. In England and Wales, this is the isolate most associated with clinical samples and the majority of environmentally isolated samples [12].

The world has been in the grips of one of, if not the greatest health challenges due to COVID-19. At the time of presentation, the UK had started lifting restrictions after a prolonged period of national lockdown. This meant that the hospitality industry, being one of the hardest hit during this period, were finally getting customers once again. The regular checks during this period of national lockdown were not possible, and with the hospitality industry essentially being completely shut down during this time, it is not unreasonable to assume water system maintenance was not carried out. At the time of writing, we are in the midst of a new variant being spread and the possibility of further lockdown restrictions being applied.

Of significance, officially published cases (with PMIDs) of concurrent Legionella and SARS-CoV-2 infection have been documented to date sporadically across Europe, Asia, and North America as a collection of letters, case reports, and series. An early study in the UK quantifying bacterial/fungal and SARS-CoV-2 coinfections had yet to identify other cases, such as ours [13]. However, a recent case series highlights that in the UK there have only been two other cases [14], which of note were also fatal. This alongside our case demonstrates a worrying trend: so far, all documented cases of Legionnaire's disease in the UK have been fatal. This could reflect the rarity of such cases and that they fall outside of the initial differential diagnosis, thus leading to delayed management, which is documented to have significantly poorer outcomes in Legionnaire's disease [15]. It could also demonstrate that strains of L. pneumophila found in the UK are more pathogenic and/or that initial treatment guidelines for COVID-19 are detrimental in such coinfections.

A case series in France identified that all cases of proven Legionnaire's disease and SARS-CoV-2 coinfection needed ICU admission in comparison to a third of those that had Legionnaire's disease without SARS-CoV-2 coinfection and also seemingly in comparison to common bacterial pneumonia pathogens and COVID-19 [16]. To our knowledge, it is the only study that had a significant number of patients with Legionnaire's disease with and without COVID-19 and allowed a comparison of outcomes. A case report of concurrent Legionnaire's disease and SARS-CoV-2 coinfection in Portugal also noted the need for ICU involvement and that, in isolation, Legionnaire's disease only required ICU in 44% of cases [17]. Most of the case reports in the US also had ICU involvement in patient care [18–20].

These cases alongside the UK cases described (ours and Chalker et al.) indicate that a possible interplay between concurrent infection with L. pneumophila and SARS-CoV-2 could be significantly different from that of other bacterial coinfections, with concurrent bacterial infection already known to lead to poorer outcomes [3]. It could also indicate that treatment for COVID-19 may in fact harm patients if they have a concurrent L. pneumophila infection, although no data is yet available and no mechanism has been put forward. A key aspect that plays an important role is the now-rapid nature of the SARS-CoV-2 test. It also indicates that these cases require early discussion and the involvement of ICU teams. Without having the full clinical picture of all other Legionella and SARS-CoV-2 coinfections, however, it is difficult to truly be able to compare our cases to those around the world. Very recent meta-analyses of Legionnaire's disease and SARS-CoV-2 coinfections [21] have identified only 18 cases across PubMed and EMBASE, which highlights the rarity but significance of Legionnaire's disease and COVID-19. We believe that our case report has expanded our knowledge as well as broadened the discussion regarding the global differences in Legionnaire's disease and SARS-CoV-2 coinfection.

It is worth bearing in mind that the UK now has a majority vaccinated population, and serious pneumonitis as a result of COVID-19 is likely to become a less likely primary cause for severe lower respiratory tract infections and pneumonia. We are in uncharted territory in this post-COVID era, as we do not know what the outcomes of giving the initial treatment for COVID-19 will be for differing bacterial coinfections and more specifically for Legionnaire's disease.

## 4. Conclusion

Our case demonstrates the clinical difficulty this brings to managing such patients before any diagnostic information is made available and the potential impact that this would have on the diagnosis' and management of lower respiratory tract infections in the post-COVID, postvaccination, and postbooster era. The world is attempting to start the “post-COVID-19” healing process, but in its haste it may be leading to more presentations such as ours. Legionella has proven itself a pathogen with anecdotally high mortality and morbidity, especially within the UK, and early discussion and admission to the ICU are warranted if there is a strong suspicion of a Legionella coinfection. Indeed, with COVID-19 having dominated healthcare for the past few years, it is not unreasonable to be blinded in some part by the confirmation heuristic. It is important to continue to practice medicine as we have been trained; with good history taking, thoroughly thoughtout investigations and maintain an open mind for differential diagnosis', these remain the cornerstone of good clinical practice with COVID-19 being part of, but not the only differential diagnosis'. We hope our case prompts a change to clinical practice and that we may be able to collaborate with authors of other clinical cases, such as ours, to create a clear protocol when managing cases of suspected Legionella and SARS-CoV-2 coinfection. Further studies are also needed to quantify if there is a true rise in mortality in concurrent Legionella and COVID-19 infection compared to either in isolation.

## Figures and Tables

**Table 1:** Laboratory investigations detailing a positive coronavirus swab test and initial hyponatremia, ^∗^ indicates abnormal results.

		Units	Values on admission	Values 2nd day of admission
Haemoglobin	(130–180)	g/L	147	140
White cell count	(4.0–11.0)	×10 9/l	10	6.2
Platelets	(150–450)	×10 9/l	225	244
Mean corpuscular volume	(76–96)	fl	95	93
Red blood cells	(4.50–5.50)	×10 12/l	4.56	^∗^4.31
Haematocrit	(0.40–0.51)	/L	0.435	0.402
Mean corpuscular haemoglobin	(27.0–32.0)	pg	^∗^32.2	^∗^32.5
Neutrophils	(2.0–8.0)	×10 9/l	^∗^9.3	5.6
Lymphocytes	(1.0–4.8)	×10 9/l	^∗^0.5	^∗^0.4
Monocytes	(0.2–0.8)	×10 9/l	^∗^0.1	^∗^0.1
Eosinophils	(0.0–0.4)	×10 9/l	0	0
Basophils	(0.0–0.2)	×10 9/l	0.1	0.1
Sodium	(133–146)	mmol/l	^∗^129	135
Potassium	(3.5–5.3)	mmol/l	4.6	4.1
Creatinine	(62–115)	umol/L	105	67
Urea	(2.5–7.8)	mmol/l	^∗^9.9	6.4
Estimated glomerular filtration rate	(60–150)	ml/min	64	96
Alkaline phosphatase	(30–130)	IU/L	58	43
Alanine aminotransferase	(10–37)	IU/L	18	18
Albumin	(35–50)	g/l	^∗^25	^∗^19
Total bilirubin	(0.0–21)	umol/l	15	9
C-reactive protein	(0.0–5)	mg/l	^∗^527	^∗^480
Ferritin	(20–275)	ug/l	^∗^631	—
Lactate dehydrogenase	(125–243)	IU/L	^∗^421	—
Troponin	(0.0–1.9)	ng/L	^∗^9.8	—
Troponin (repeat at 3 hours)	(0.0–1.9)	ng/L	^∗^8.1	
D-dimer	(0.0–500)	ng/ml	^∗^7088	—
Procalcitonin	(0.0–0.5)	ug/L	^∗^6.8	—
Coronavirus polymerase chain reaction			Positive	Positive
